# New Structural Patterns in Moribund Grammar: Case Marking in Heritage German

**DOI:** 10.3389/fpsyg.2015.01716

**Published:** 2015-11-20

**Authors:** Lisa Yager, Nora Hellmold, Hyoun-A Joo, Michael T. Putnam, Eleonora Rossi, Catherine Stafford, Joseph Salmons

**Affiliations:** ^1^Center for the Study of Upper Midwestern Cultures, University of Wisconsin–MadisonMadison, WI, USA; ^2^Department of Germanic & Slavic Languages and Literatures, Pennsylvania State University, University ParkPA, USA; ^3^Psychology and Sociology Department, California State Polytechnic University, PomonaCA, USA

**Keywords:** bilingualism, heritage language, reanalysis, case marking, case syncretism, differential object marking, German

## Abstract

Research treats divergences between monolingual and heritage grammars in terms of performance—‘L1 attrition,’ e.g., lexical retrieval—or competence—‘incomplete acquisition’, e.g., lack of overt tense markers (e.g., Polinsky, 1995; Sorace, 2004; Montrul, 2008; Schmid, 2010). One classic difference between monolingual and Heritage German is reduction in morphological case in the latter, especially loss of dative marking. Our evidence from several Heritage German varieties suggests that speakers have not merely lost case, but rather developed innovative structures to mark it. More specifically, Heritage German speakers produce dative forms in line with established patterns of Differential Object Marking (Bossong, 1985, 1991; Aissen, 2003), suggesting a reallocated mapping of case. We take this as evidence for innovative reanalysis in heritage grammars (Putnam and Sánchez, 2013). Following Kamp and Reyle (1993) and Wechsler (2011, 2014), the dative adopts a more indexical discourse function, forging a tighter connection between morphosyntax and semantic properties. Moribund grammars deploy linguistic resources in novel ways, a finding which can help move us beyond simple narratives of ‘attrition’ and ‘incomplete acquisition.’

## Introduction

Most research on the grammar of bilinguals known as ‘heritage speakers’ is framed in terms of what speakers cannot (or can no longer) do, compared to monolingual speakers of their heritage languages, and research typically accounts for these deficiencies in terms of ‘incomplete acquisition’ and/or ‘attrition’ (e.g., Benmamoun et al., 2013 and responses to them in the same journal issue). For instance, Montrul et al. (2015, p. 567) summarize research to date as showing that (emphasis added):

Inflectional morphology, semantics, and the syntax–discourse interface are quite vulnerable to simplification and loss. Several studies of different heritage languages that used different methodologies have shown that HERITAGE SPEAKERS DO NOT MASTER CASE ...

Here, we seek to reorient discussions away from that focus on lack or loss and toward understanding heritage grammars in terms of active reanalysis, in line with some other work on early bilinguals (e.g., Kupisch and Barton, 2013) as well as similar arguments made for non-sequential bilinguals across the lifespan (Putnam and Sánchez, 2013). We reinterpret a classic example of ‘loss’ in a heritage grammar as an innovative reanalysis on the part of heritage speakers. That example is ‘case’ in diasporic varieties of German. Many German varieties have three nominal cases (nominative, accusative, dative) and one common scenario is that morphological dative, historically present across Germanic and still present in Standard German (SG) and other varieties, appears to be lost, leaving a nominative-oblique system. This shift has happened in European varieties and heritage varieties. It is exemplified in (1), from Wisconsin Heritage German:

**Table:** (1) Wisconsin Heritage German (WHG) case marking

(a) Standard-like dative
WHG	Standard German
im Boom	im Baum
in-the-_DAT_ tree	in-the-_DAT_ tree
(b) Accusative for SG dative
von ein Dorf	von einem Dorf
from a-_NOM-ACC_ village	from a-_DAT_ village
(c) Innovative marking
es war in den Haus	im Haus
it was in the-_ACC_ house	in the-_DAT_ house

Example (1a) reflects that dative is not entirely lost in these varieties, while (1b) exemplifies a morphologically ambiguous form, presumably an accusative in this context, though surface-identical with the nominative form for neuters. As shown in (1c), we also find some innovative marking, in this case a form, *den*, that would be distinctly accusative for a masculine but used here with a neuter noun, which would show no distinction in the standard, as just noted.

Patterns of case reduction have also been observed in other heritage languages (e.g., Russian in Polinsky, 1995, Hindi in Montrul et al., 2012, and comparatively across Spanish, Hindi, and Romanian in Montrul et al., 2015). We present data from three different contact settings and five German varieties in total that show dative marking that differs from canonical three-case systems.

Previous analyses have treated such changes both in terms of failure to acquire case morphology and/or loss through attrition. ‘Incomplete acquisition’ (Montrul, 2008), understood essentially as the arrested development of certain features of the heritage language (see below), is an unlikely culprit in this process since most speakers in the present study were monolingual speakers of German until around age six, well after when dative would have normally been learned, around age three (Eisenbeiss et al., 2009). Attrition, taken as the loss of some structural property after it has been successfully acquired, would then seem like the obvious source of case loss.

However, closer analysis suggests a more nuanced view, namely, that speakers are developing patterns of Differential Object Marking (DOM), following a hierarchy in which preferences are shown cross-linguistically for marking case on animate and definite arguments over inanimate and indefinite ones. Aissen (2003, p. 435) defines it this way: “It is common for languages with overt case-marking of direct objects to mark some objects, but not others, depending on semantic and pragmatic features of the object.” In the literature, DOM effects are often expressly restricted to DIRECT objects, though the literature since Bossong (1991) has treated complex interactions involving dative objects. As Aissen (2003, p. 446) writes, “In a number of the languages ..., accusative case in a DOM system is identical to dative case ...” In Spanish, for instance, the DOM marker, ‘personal *a*,’ is also used for indirect objects, and in Hindi *-ko* marks DOM on direct objects but also indirect objects (Montrul et al., 2015, p. 570). Here, dative case marking is retained more often on pronouns than on determiners and, in some varieties, more on definites than indefinites. On the empirical side, this is the first time to our knowledge that the EMERGENCE of new DOM effects has been described for heritage languages. More detailed discussion of dative DOM is left for future work.

Changes in morphological case marking, based on these results, should not simply be viewed as a loss of inflectional morphology but rather need to include the emergence of new semantic-morphosyntactic mapping strategies. Our general conclusion is that heritage bilingual grammars are complete grammatical systems that show structural innovations of the sort we expect in any living language. The patterns we observe are understandable in terms of reanalysis of structural systems (e.g., Polinsky, 2011; Putnam and Sánchez, 2013), and this discussion begins to move research toward modeling the actual implementation.

The question of whether particular ‘vulnerable domains’ exist in developing bilingual grammars has been pursued in previous studies (e.g., Paradis and Genesee, 1996; Hulk and Müller, 2000; Meisel, 2001; Müller and Hulk, 2001). A primary focus of this research has been on whether or not some aspects of morphosyntax may be affected by interdependent developments rather than the entire grammar system. The general consensus argues for interdependence primarily except for when the grammar interacts with other cognitive (i.e., extra-grammatical) interfaces.

The rest of this paper is structured as follows. The next section gives a brief overview of German case and apparent case reductions in heritage German (‘speech islands’) and for Germanic more generally. §2 introduces ‘incomplete acquisition’ and ‘attrition’ as they have been applied to reductions in inflectional morphology among heritage language speakers, along with data on L1 German case acquisition. §3 presents methods and data from a set of heritage German varieties: §3.1 for Texas German, §3.2 for three varieties from Wisconsin and §3.3 for some initial data on Misionero German (MG) from South America. §4 concludes.

## Case Marking and Case Reduction in Germanic and Heritage German

While SG has a four-case system, the genitive is not widely used in colloquial varieties either historically or today; moreover, genitive case was likely present in heritage varieties only through exposure in school or reading formal texts for most, so that discussion of Heritage German case best starts from a three-case system, consisting of nominative, accusative, and dative.^1^ Case is marked on many pronominal forms and on determiners, though there is considerable syncretism in some paradigms. **Table 1** shows examples of three pronominal and three definite article paradigms drawing on two of German’s three genders, masculine and feminine.

**Table 1:** Example nominal paradigms for German case.

	Pronouns			Determiners		
	1 sg.	2 sg.	3 fem. sg.	masc. sg.	fem. sg.	Plural
Nominativ	ich	du	sie	der	die	die
Accusative	mich	dich	sie	den	die	die
Dative	mir	dir	ihr	dem	der	den(en)

The distinction between structural and lexical case in German is debated and here we follow Eisenbeiss et al. (2009, pp. 9–10), who treat accusatives (as either direct objects or complements of prepositions) and datives in the function of indirect object as structural. Dative forms appearing as complements of prepositions or with verbs that govern the dative (*helfen* ‘to help’, *antworten* ‘to answer’) are considered lexical. As reviewed by Eisenbeiss et al. (2009), alternatives and variants include views that treat all datives as lexical (Haider, 1985; Haegeman, 1991), that treat all prepositional case use as lexical (Haegeman, 1991; Heinz and Matiasek, 1994), and that treat prepositional datives as structural and accusatives as lexical (Bierwisch, 1988).

**Table:** (2) Structural vs. lexical case, after Eisenbeiss et al. (2009), focusing on datives

** Structural**
Nominative and accusative on direct objects.
**ich** glaube ‘I believe’, **sie** arbeitet ‘she works’
sie sieht **mich** ‘she sees me’, wir kennen **den** Mann ‘we know the man’
Dative on indirect objects:
er gibt es **denen** ‘he gives it to them’, sag **mir** etwas ‘tell me something’
** Lexical**
Dative with complements of prepositions:
mit **mir** ‘with me’, nach **dem** Film ‘after the movie’
Dative with ‘2Prep’^2^ (locative)
in **der** Schule sein ‘to be in school’, auf **dem** Bett liegen ‘to lay on the bed’
Dative with ‘dative verbs’:
hilf **mir** ‘help me’, gehört **ihr** ‘belongs to her’

Transitive verbs that govern the dative require an object in dative case. This means that the case of the direct object is item-based and not structural. In contrast, ditransitive verbs require a direct object in accusative case and an indirect object in dative case. A simple transformation task illustrates the difference:

**Table:** (3) The syntactic distinctiveness of ‘dative verbs’

(a)	Ich	sehe den	Mann.	Der	Mann wird gesehen.
	I-_NOM_	see the-_ACC_	man	the-_NOM_	man is seen
(b)	Ich	helfe dem	Mann.	Dem	Mann wird geholfen.
	I-_NOM_	help the-_DAT_	man	the-_DAT_	man is helped
				*Der	Mann wird geholfen.
				the-_NOM_	man is helped

In the case of a verb that governs the dative the direct object cannot be promoted to the subject position in a passive sentence.

A cross-linguistically common pattern of case marking is DOM. Following Aissen (2003), DOM occurs in languages with overt case marking where some direct objects are marked and others are not. What governs DOM is dependent on semantic and pragmatic contexts. Though DOM has not been widely discussed for Germanic, the phenomenon has been the focus of numerous functional, formal, and hybrid perspectives (Lazard, 1984; Bossong, 1985, 1991; de Hoop, 1992; Aissen, 2003; Naess, 2004; de Swart, 2007; Dalrymple and Nikolaeva, 2011). Dalrymple and Nikolaeva (2011, p. 2) argue that “marked objects are associated with the information-structure role of **topic**. The association may be either synchronic or historical. Where the direct connection between marked objects and topicality has been lost through grammaticalization, marked objects in some languages become associated with **semantic features** typical of topics (animacy, definiteness, specificity).” While many architectural and operational differences exist across contemporary linguistic formalisms, we adopt Dalrymple and Nikolaeva’s position.

It has often been observed that pronouns retain dative markings longer than determiners or noun phrases, e.g., in the history of English (Lass, 1992, p. 140ff.), but the same pattern is found across various languages undergoing case loss, including Romance, where Spanish, French, and Italian no longer show case in noun phrases but typically retain nominative-oblique and often other forms in pronouns (e.g., Spanish first singular *yo, me, mío(s)/mía(s), mí, conmigo*). For diasporic varieties of German, Rosenberg (2005, p. 230) describes things this way:

German-speaking language islands also share another striking feature which may result from an internal typological drift common to all German varieties or even to all Germanic and other Indo-European languages: while case reduction in the nominal paradigms is extensive, it is not in the pronominal paradigms. Personal pronouns frequently have a three-case system or retain at least the dative, which includes the possibility of marking the direct-indirect object relation (by common case vs. dative).

This retention of dative marking on pronouns over determiners has been accepted as a pattern, but not placed in a broader context. DOM effects, we propose, play a very different role in Heritage German: Ostensible loss of dative can be better seen as reanalysis of old morphological/syntactic case marking into a new system of variable DOM. DOM has, in fact, been described as “syntactic rules conditioned by semantic factors” (Baerman, 2008, p. 229). None of the long tradition of diachronic research on Germanic case reductions just mentioned discusses DOM and case loss at all to our knowledge. If there are DOM effects in Heritage German realizations of the SG dative, this leads to some easily testable predictions:

• Pronouns should show case marking over full NPs, e.g., *mit **mir*** but *mit **den** Mann*;
• Definite should show case marking over indefinite, e.g., less dative on *ein*-determiners (indefinite) than *der*-determiners (definite), so standard *einem* should be realized as *ein/einen* more often than *dem* as *der/den* or *das.*
• Animate should show case marking over inanimate, so humans and animals should show more dative determiners than physical objects.

The ongoing historical loss of morphological case in Germanic languages is reconstructible since the transition from Indo-European to Proto-Germanic. It has been intensely studied for decades from almost every conceivable perspective (see Bousquette and Salmons, forthcoming, or specifically on German, Salmons, 2012). Diasporic German dialects, ‘language islands,’ show especially widespread patterns of case change, especially dative. This is reported for varieties spoken in Eastern Europe, Brazil, Australia, South Africa, and across North America (see, among many others, Rosenberg, 1994, 2005; Nützel and Salmons, 2011).

Barðdal and Kulikov (2008, p. 470) review various scenarios for case reduction, including phonetic-phonological, morphological and syntactic-semantic accounts, noting that case loss is “typically preceded by a period of variation and alternation between case forms or argument structures.” Language contact clearly correlates with loss of inflectional morphology (O’Neil, 1978; Maitz and Németh, 2014). This is one of the most robust findings across myriad dialects and contact settings for heritage German varieties. And as already noted, the pattern extends far beyond Germanic. Benmamoun et al. (2013, p. 142) state: “Morphological deficits in heritage languages are asymmetric; they seem to be more pronounced and pervasive in nominal morphology than in verbal morphology.” We turn now to the two major accounts of this pattern.

## Explaining Reduction of Inflectional Morphology: Incomplete Acquisition and Attrition

As previously noted, the two main accounts of morphological reduction in heritage grammars involve incomplete acquisition and attrition. We treat each in turn after a word about the acquisition of case.

The basic picture of how functionally monolingual L1 learners acquire case proceeds as follows, according to Mills’ (1985, p. 155) classic study (confirmed by much research since, which we will not review here):

The marking of case in the nominative and accusative is only apparent in the masculine gender paradigm. The distinctive marking of nominative and accusative is sporadic before age 3;0; otherwise the nominative case form is used. This can probably be attributed to an attempt to regularize the paradigm since in the feminine, neuter, and plural paradigms there is no distinction. Dative case appears around age 3;0 and is usually marked correctly except after prepositions. Genitive case does not appear marked on the article in any of the data reported ... .

Prepositions start to appear regularly, predominantly in locative use, around age 3;0. Accusative case is frequently overgeneralized after prepositions. This is probably due to the easy confusion of *n* (marking accusative) and *m* (marking dative) in the masculine gender paradigm. From experimental evidence the stative meaning appears to be learned before directional meaning with those prepositions which can have both meanings.

Eisenbeiss et al. (2009) compare two groups of children, a set of typically developing (TD) children and a set of children with Specific Language Impairment (SLI), the former aged 2;6-3;6 at the time of recording and the latter 5;8-7;11. For both groups, structural case was highly accurate and lexical datives, either with prepositions or verbs, were about half dative and half accusative. They also note that case marking is often omitted on what they call ‘*ein*-determiners’: indefinite articles, possessive pronouns, and the negation element *kein*- ‘no’. We will pick up on this again below.

Turning now to incomplete acquisition, it is a concept that receives much attention but which often remains ill-defined and poorly understood. Montrul (2008, p. 21), whose treatment of this topic is perhaps the most detailed available, understands incomplete acquisition as “(for lack of a better term) ... a mature linguistic state, the outcome of language acquisition that is not complete or attrition in childhood. Incomplete L1 acquisition occurs in childhood, when, for different reasons, some specific properties of the language do not have a chance to reach age-appropriate levels of proficiency after intense exposure to the L2 begins.” According to this definition, language acquisition is truncated—incomplete—in bilingual speakers whose developing L1 grammar receives insufficient input (from the standpoint of quantity and/or quality of input) during the formative earlier years of language acquisition (i.e., prior to puberty for Montrul, but see Paradis, 2009 on dating it much earlier, to 2–5 years). The concern is reinforced by Meisel et al. (2013, p. 149) that “the notion of ‘incomplete acquisition’ is not defined with the desirable precision in the literature on heritage languages.” Other views exist, such as those of Pascual y Cabo and Rothman (2012) and Putnam and Sánchez (2013), that heritage grammars are completely acquired grammars, yet distinct from those of other monolingual and bilingual speakers.

It is very unlikely that the emergence of DOM effects in the varieties of diasporic Heritage German we investigate here stems from insufficient input during L1 acquisition or an inability of the speakers to convert this input into intake such that it is integrated into the developing grammar.

Bentz and Winter (2013, p. 18) argue that languages with more L2 speakers, i.e., languages that are used by many speakers who have learned them as a second language, show more case loss than languages with fewer L2 speakers. This fits with evidence that L2 acquisition of case is difficult even under the best of circumstances. They extend their discussion to language ‘enclaves,’ using an example from a variety of Heritage German, the one which will provide our first case study below:

a common finding is that inflectional paradigms are maintained in the first generations after immigration, but in the following generations morphological systems are quickly simplified ... For example, in Texas German, use of the dative went down from 64 to 28.5% (Salmons, 1994, p. 61) within only one generation. This dramatic change happened when ... a considerable number of parents (Boas, 2009, p. 349) decided not to speak Texas German with their children. Thus, the children of this variety successively became L1 speakers of English and L2 learners of Texas German ... This opens up the possibility that case loss is at least partly due to imperfect L2 learning.

Boas does not actually claim that the last generation of Texas German speakers was L2 learners. L2 learning of heritage varieties is rare, and this view seems to reflect basic misunderstandings about heritage languages (cf. Rothman and Treffers-Daller, 2014). Salmons (1994) actually associates the decline in dative marking with the loss of exposure to SG when schools switched from German- to English-medium instruction.

In addition to incomplete acquisition, much literature centers on L1 attrition, referring to a decline in performance-based (vs. competence-based) attributes of a grammar that have been completely acquired. As Montrul (2008, p. 65) clarifies, “attrition in adults affects primarily performance (retrieval, processing, and speed), but does not result in incomplete or divergent grammatical representations.” This definition is more or less consistent with other definitions of attrition, such as the one provided by the Oxford English Dictionary (OED Online): “the gradual disappearance of a linguistic feature from a language. Later also: the gradual decline in use of or loss of ability in a language, esp. in a bilingual or multilingual community.” With that background, we now turn to data from three varieties of Heritage German which can then be considered in the terms of this discussion.

## Data From Heritage German

This section illustrates variable realizations of accusative and especially dative forms across several varieties and regions: in Texas German, in three varieties spoken in eastern Wisconsin, and in a variety of German spoken in South America. ‘Dative loss’ has often been treated in the black-and-white terms that the name suggests. The first dataset is a reanalysis of old data, while the second comes from work in progress and first reported here and the third set is a first exploration undertaken specifically for this project. Note that these are not the typical heritage speakers discussed in recent research, but instead bilinguals whose families have been, as described below, speaking German varieties in societies with other dominant languages for several generations.

We begin to add some nuance here, first in relatively familiar ways, like realization of dative on pronouns vs. full noun phrases, but then extending to definiteness and animacy.

### Texas German

As described in many works, most extensively in Gilbert (1972), German speakers settled in especially central Texas. The settlement was chronologically relatively compact, starting in the 1840s and the language was transmitted over generations until the late 20th century.

Salmons (1994) provides an analysis of Texas German data, based on Gilbert’s (1972) *Atlas*, where a set of sentence translations involved what would be SG dative forms, e.g., ‘he came with me’, cf. SG *mit mir* (dative) and ‘he’s already in the room,’ Standard *im Zimmer*. The first point was to establish that the dative had in fact once been widespread in Texas. **Table 2** below presents that data, showing a rapid and sharp decline in the use of dative among Gilbert’s consultants born after about 1911, which Salmons attributes to the removal of German as the medium of instruction in schools around that time.

**Table 2:** Texas German dative vs. accusative for standard dative, regional/age stratification, from Salmons (1994).

Date of birth	NW	WC	SW	NE	Total	Percentage
–1899	20–13	43–29	52–16	29–16	144–74	66.1
1900–1911	21–17	22–15	21–11	17–23	81–66	55.1
1912–	16–60	4–30	20–21	9–12	49–123	28.5

*(Regions: NW = Northwest, WC = West Central, SW = Southwest, NE = Northeast).*

Further analyses in Salmons (1994) were focused on speakers from particular regions. **Table 3** presents the numbers there, rearranged for our purposes to capture Eisenbeiss et al.’s (2009) distinction between lexical and structural case, discussed above. While Eisenbeiss et al. (2009) found that L1 acquirers mastered structural case quickly and lexical case only later, Texas German adults do not show parallel patterns: The lowest rates of dative are found with prepositions that can either govern dative or accusative, depending on whether they involve location (dative) or motion across a boundary (accusative).

**Table 3:** Dative by context (raw numbers).

	Struct.	Lexical, prep		Lexical, 2prep			Lexical, verbal	
	gib ihr ‘give her’	mit mir ‘with me’	mit ihr ‘with her’	über dem Bett ‘above the bed’	unter dem Baum ‘underneath the tree’	im Zimmer ‘in the room’	hilft mir ‘helps me’	gehört ihnen ‘belongs to hem’
Dative	23	22	15	3	5	6	12	34
Accusative	38	36	23	41	30	22	50	24

The clearest correlate of where dative is or is not marked is in fact what element it is marked on. As shown in **Table 4**, use with determiners was strikingly low compared to use with pronouns.

**Table 4:** Case use with ...

	Pro		Det	
Dative	104	35.4%	14	8.3%
Accusative	175	59.5%	150	88.8%

The distinction between lexical and structural case, the observations from which this study ultimately grows, is suggestive of the DOM patterns discussed above, where pronouns are at the top of the DOM hierarchy. Let us turn to data from Wisconsin.

### Three Wisconsin Communities

A large number of German-speaking settlers arrived in Wisconsin in the latter two-thirds of the 19th century. Unlike the settlement patterns for Texas German, in Wisconsin immigrants from similar geographic, cultural, and linguistic backgrounds often settled together in communities due to social contacts and shared backgrounds, which prevented some contact and supported relatively closed social networks (Frey, 2013, pp. 119–120, and elsewhere). SG also played a role in these communities; members were often fluent in both a dialect and a kind of High German, mutually intelligible with the standard language. The speech of Wisconsin Heritage German (WHG) speakers today can be described as a standard-like koiné with dialect features.

Yager (forthcoming) compares case marking on nominal and pronominal tokens by 21 WHG speakers from three distinct communities in eastern Wisconsin. Noun phrases and personal pronouns from semi-structured interviews^3^ were categorized and coded based on set characteristics, e.g., gender, number, case, article type, animacy, etc. A total of 5,191 nominal and pronominal tokens were analyzed.

The consultants all learned a German koiné at home as their L1, as described above, and acquired English, typically when they began school. They come from three adjacent but distinct regions in eastern Wisconsin (with seven speakers from each region), which are represented by communities with common social networks and settlement histories. The region known as the Holyland was settled by Catholic immigrants from the Eifel region in western central Germany. Lutherans from Rheinhessen settled in the city of Sheboygan and the surrounding area, while the region around the town of Kiel was settled by Low German speakers. Each of these German dialectal regions is known to deal with the German case marking system in different ways, ranging from a three-case system in Rheinhessen, to a nominative-oblique two-case system in the Eifel region, to a single-case system for nouns in Low German dialects.

**FIGURE 1:**
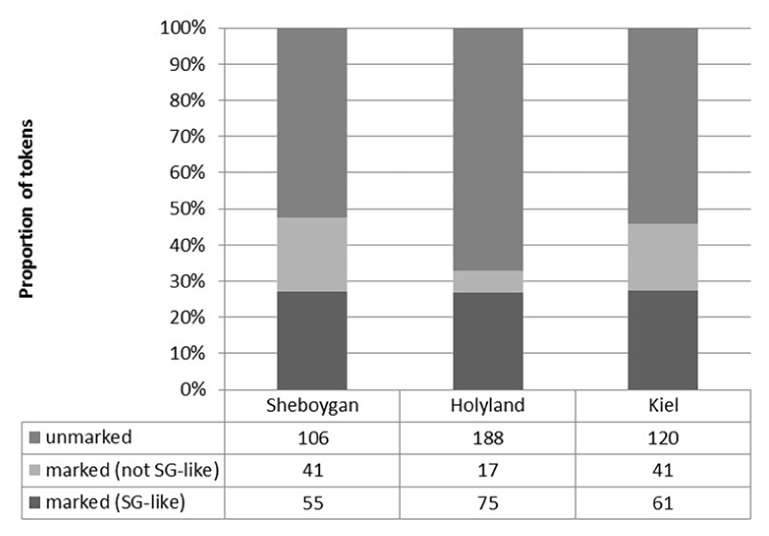
**Wisconsin German case marking by region (definite NPs)**.

Although the settlement histories and baseline dialects vary across the three communities, each group appears to mark case in similar ways, illustrated already in (1) at the outset of this article. **Figure 1** shows the proportion of case-marking on definite NPs by region.^4^ Each group produces a similar proportion of SG-like case marking versus non-SG-like case marking, i.e., where an object determiner shows a case-marked form that would not be expected, e.g., for the accusative feminine article, which would be identical to the nominative article in SG. The differences in case marking between each of these groups are not statistically significant.^5^

**FIGURE 2:**
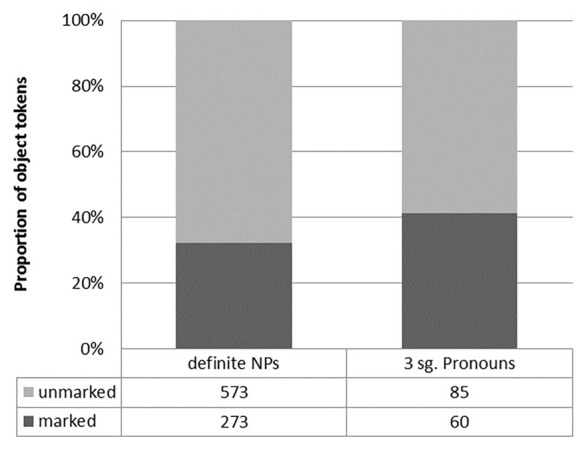
**Differences in Wisconsin German case marking between NPs and pronouns**.

With DOM, we would expect to find a higher frequency of case marking on pronouns compared to NPs, as pronouns tend to show a greater degree of both definiteness and animacy. **Figure 2** illustrates these findings for WHG. As **Figure 2** shows, 32.2% of oblique definite NPs are marked in some way, while third person singular pronouns show marking on 41.4% of all tokens. The difference between these two proportions is statistically significant. The overall higher degree of case marking on pronominal tokens is in line with DOM.

There was no significant difference between case marking on animate versus inanimate NPs. However, definite NPs did show a higher frequency of case marking than indefinite NPs. **Table 5** compares case marking on masculine definite and indefinite tokens.

**Table 5:** Wisconsin German case and definiteness.

	Marked	Unmarked	Total tokens
Definite accusative	70	81	151
Indefinite accusative	1	27	28
Definite dative	127	32	159
Indefinite dative	0	3	3

Although the numbers of indefinite tokens are low, the lack of marked indefinite forms compared to the proportion of marked definite forms suggests a correlation between definiteness and case marking in line with DOM.

Not only is the SG case-marking system retained to some extent in each of the three WHG communities, there also appears to be a restructuring of the system around semantic principles, reflecting the emergence of DOM effects.

### Misionero German

Misionero German comprises regional dialects of German from the Volga German area spoken in the Misiones province in northeastern Argentina. MG speakers acquired the German variety as their first language (L1). Over time, they have become dominant in their L2 Brazilian Portuguese, the current language of the community, and MG has become moribund. Later, these MG speakers, especially those under the age of 40, acquired Spanish as an L3, which is also widely spoken throughout the Misiones Province. Today, the majority of these transitional trilingual German-Portuguese–Spanish speakers are settled along the upper part of the Uruguay River, from El Soberbio to Panambí. The following data come from speakers in this region (see Putnam and Lipski, forthcoming for an overview).^6^

**FIGURE 3:**
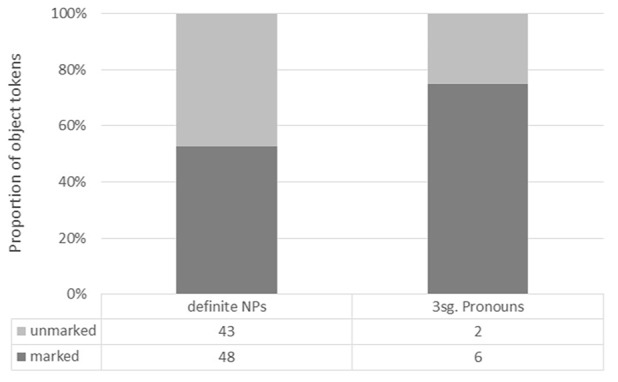
**Differential Object Marking (DOM) in full NPs and third person singular pronouns in Misionero German (MG)**.

Free speech data from seven speakers were transcribed and analyzed following the conventions used in Yager (forthcoming), yielding a total of 1,565 tokens; 842 in NP; 283 of these in PP; and 697 pronouns. Because the raw numbers for this first sample are extremely low, not allowing even for use of non-parametric statistics, we report results as descriptive statistics, which will allow describing a general trend in the pattern of performance. The addition of more data in the next phases of this research will be important to confirm the observed trends. First, we looked at differences in case marking between full NPs and pronouns in order to analyze the data for possible DOM effects, as reported in **Figure 3**. Even though the overall number of third person singular pronouns is very small compared to the definite NPs, these preliminary results show that pronouns tend to be marked more frequently (75%) than NPs (53%).

Second, we looked at differences between case marking in definite and indefinite determiners. Even with a small number of tokens, a trend can be seen toward more case marking on definite than indefinite determiners. These results are summarized in **Table 6**. For accusative case marking, only 55% of definite determiners are case-marked. However, 80% of indefinite determiners are unmarked for case. Against this background, a slight trend for DOM of definite determiners can be inferred. Dative case marking shows a similar pattern, with 59% of definite determiners being case-marked.

**Table 6:** Trend toward DOM in definite and indefinite determiners in Misionero German.

	Marked	Unmarked	Total
Definite accusative	5 (55%)	4 (45%)	9
Indefinite accusative	1 (20%)	4 (80%)	5
Definite dative	16 (59%)	11 (41%)	27
Indefinite dative	1 (50%)	1 (50%)	2

No DOM effect was found for animate versus inanimate objects. The analysis of the MG data shows DOM with pronouns more marked for case than full NPs, and definite determiners more than indefinite ones. These findings align with the results from WHG.

In summary, one of the most widespread findings in diasporic German has been the loss of case, especially dative marking. Taking a different approach where we examine more nuanced patterns of the realization of dative, a different picture emerges: Across Texas German, three varieties of Wisconsin German and Misionero German, we find distinct but related patterns of case marking, all consistent with dative-based DOM effects.

## Conclusion

The data presented here point to the emergence of a cross-linguistically familiar generalization in the realization of case marking, namely a particular form of DOM. Traditionally framed in terms of loss or attrition, these patterns in fact show the development of new grammatical generalizations in these communities. Our findings complicate the traditional narrative of loss and simplification in heritage language grammar, especially with regard to nominal morphology.

The communities analyzed here are geographically very distant from one another, and in contact with different, typologically distinct languages and dialects. In their comparative study of DOM-loss in the English-dominant context of North America, Montrul et al. (2015, p. 566) observe that heritage speakers of Spanish, Hindi, and Romanian “seem to adopt the grammar of English, which does not overtly mark direct objects, and accept non-target sentences with animate, specific direct objects without DOM.” The patterns observed in our data, though, cannot be explained simply in terms of direct influence from sociolinguistically dominant L2 grammars, i.e., English, rural vernacular Portuguese, and Spanish. Nor can they reflect spread from one community to another, and because the original input varieties were from different areas and German does not show classic patterns of DOM effects, they are very unlikely to have sprung from seeds imported with initial immigration. Instead, we see a new, divergent grammatical property, the rise of DOM. As is often the case with DOM, its occurrence is tendential rather than categorical.

Appealing to incomplete L1 acquisition as the force behind these changes is not promising, because, as we have noted, German-speaking children develop command of structural case by age 3. We thus should expect children exposed until school age to varieties of German that license dative case to have successfully acquired at least structural datives. All speakers use the dative in a range of grammatical contexts (both structural and lexical), including those with more or less exposure to SG. Similarly, L1 attrition is unlikely since the DOM-patterns we observe are arguably as complex as or more complex than the earlier system. To understand these patterns, we must get past the narratives of “collapse” and “loss” that are commonly attributed to heritage grammars.

In contrast, the patterns we find here are consistent with the position of Putnam and Sánchez (2013), who see heritage grammars as full grammars, capable of change, including reanalysis, in the ways that all grammars are. At the same time, our results also raise issues to be pursued in later work. For instance, how do typological drift and ease-of-processing procedures inform the restructuring process (cf. Hawkins, 2004; Culicover, 2013)? Another challenge regards the connection between more structural units such as morphology and syntax and their relationship to semantics and pragmatics/information structure (see §2.3). Also, our work suggests that variability in heritage grammars should include factors such as age of the speakers, specifically vis-à-vis cognitive functions. Language performance changes with normal aging, as a factor of cognitive changes that occur in normal aging. As Rossi and Diaz (forthcoming) point out, language changes due to normal aging are at times conflated with changes in language processing due to bilingualism and language contact. The populations that were tested in this set of studies exemplify how investigating heritage languages in speakers at different ages (younger adults and older adults) are of importance for future research.

A final question is whether these observed trends occur more broadly across Germanic, past and present. It would be a worthwhile pursuit to explore whether other Germanic languages that have lost case also reorganize their inflectional systems along similar lines.

## Conflict of Interest Statement

The authors declare that the research was conducted in the absence of any commercial or financial relationships that could be construed as a potential conflict of interest.

## Footnotes

1 We leave aside here varieties that have only two cases, e.g., most dialects of Low German.

2 We use ‘2Prep’ to refer to prepositions that govern dative or accusative, the former for locative and the latter for motion across boundaries.

3 The interviews, which took place between 2011 and 2014, were conducted by a group of researchers including Alyson Sewell and transcribed by Alyson Sewell. The interviews were carried out in accordance with the requirements and with the approval of the Institutional Review Board of the University of Wisconsin–Madison, under the protocol “Germanic languages and dialects in Wisconsin” (2013-1639).

4 Two consultants from the Holyland were excluded from the table because they did not produce any case-marked NPs.

5 Unless otherwise noted, tests of statistical significance are calculated with a two-tailed *Z* test for two population proportions where *p* = 0.05.

6 The interviews, which took place in the summer of 2012, were conducted by John Lipski and Michael Putnam. The interviews were carried out in accordance with the requirements and with the approval of the Institutional Review Board of Penn State University, under the protocol “Argentina-language contacts” (PRAMS: 00040019).
